# Organized after‐school activities and Asian American high school students' Asian American identity and Asian American racial identity ideological values

**DOI:** 10.1111/jora.70249

**Published:** 2026-08-21

**Authors:** Fuko Kiyama, Sandra D. Simpkins

**Affiliations:** ^1^ School of Education University of California Irvine California USA

**Keywords:** Asian American adolescent development, ethnic–racial socialization, identity exploration, organized activities, qualitative

## Abstract

The present study qualitatively examined how Asian American college students thought their participation in organized after‐school activities during high school was related to their Asian American identity and Asian American racial identity ideological values. Semi‐structured qualitative interviews were conducted with 25 Asian American‐identifying undergraduate students (80% female, 96% U.S.‐born) enrolled in a 4‐year, public university in Southern California. Thematic analysis, employing both inductive and deductive approaches, identified two key mechanisms through which Asian American college students thought organized activities helped them understand their Asian American identity: (1) exposure to same and different ethnic–racial peers and activity staff, and (2) ethnic–racial socialization. Additionally, students identified that some activities supported their engagement with Asian American racial identity ideological values—Asian American unity, interracial solidarity, and transnational critical consciousness—through peer interactions and intentional activity content. Findings highlight how organized activities may serve as meaningful spaces for ethnic–racial identity development among Asian American college students, including their engagement with Asian American racial identity ideological values. The current findings not only contribute to theories on ethnic–racial socialization but also have practical implications for organized activities of all types to support and affirm Asian American college students' ethnic–racial identity and identity ideological values.

## INTRODUCTION

Ethnic and racial identity is important for the normative development of adolescents of color in the United States (Lee Williams et al., 2012). Within the broader framework of ethnic–racial identity (Umaña‐Taylor et al., 2014), racial identity status alongside ethnic identity affirmation and belonging have been linked to positive developmental outcomes (e.g., psychological well‐being and social adaptation) for Asian American adolescents (Anyon et al., 2014; Iwamoto & Liu, 2010). Although research on ethnic–racial identity development among Asian American adolescents has grown, much of it has focused on ethnic identity, particularly through the lens of acculturation, with less attention to the specific experiences of racialization that also shape Asian American's identity (e.g., dealing with the model minority stereotype and being positioned as perpetual foreigners, Juang et al., 2017; Rivas‐Drake et al., 2014; Saavedra & Yoo, 2023). The development of Asian American racial identity ideological values (AARIIV)—values reflecting the shared racialized experiences of Asian Americans—positions racial identity as a core aspect of Asian American's identity as it provides a foundation for fostering social change‐oriented outcomes, such as critical consciousness and collective action (Yoo et al., 2021). Scholars have argued that there is a pressing need to examine what shapes Asian American adolescents' racial identity ideological values (Juang et al., 2017).

Organized after‐school activities have long been recognized as vital spaces for Asian American adolescents' positive development (Chang & Shih, 2021). Prior research on racially/ethnically diverse adolescents highlights that community‐based organized activities focused specifically on race and ethnicity can support ethnic–racial identity development (Kiyama et al., 2026). However, this work provides little insight into identity processes in organized activities for Asian American adolescents, including their AARIIV, which are critical for understanding how activities can holistically support identity development (Yoo et al., 2021). Furthermore, less is known about these processes in a broad range of organized after‐school activities, including those without an explicit ethnic–racial focus (e.g., athletic team, academic club without an ethnic–racial focus). Organized after‐school activities include structured, adult‐supervised, and goal‐directed activities occurring during the academic year (Vandell et al., 2015). Organized after‐school activities that fall under this definition include school‐based extracurricular activities (e.g., school‐based clubs, student council, sports), general after‐school programs, and community‐based organized activities outside of the regular school day. This definition excludes intensive time‐limited programs such as summer camps, which differ in structure and duration from the organized after‐school activities defined above (Vandell et al., 2015). To address this gap, the present study qualitatively explored how Asian American college students thought their participation in organized activities during high school shaped their understanding of their Asian American identity and, specifically, how these activities supported their engagement with AARIIV.

### Ethnic–racial identity and AARIIV

The ethnic and racial identity in the 21st‐century group (Umaña‐Taylor et al., 2014) proposed a multidimensional framework of ethnic–racial identity, defined as a meta‐construct reflecting the beliefs and attitudes that individuals have about their ethnicity and race (Umaña‐Taylor et al., 2014). Although ethnic–racial identity is a key developmental outcome among Asian Americans, scholars have critiqued the field's overreliance on acculturation frameworks that prioritize ethnic identity while overlooking the development of racial identity that reflects Asian Americans' experiences of racialization (e.g., model minority stereotype, perpetual foreigner, Juang et al., 2017).

Racialization refers to the social processes through which individuals are assigned a racial identity and positioned within racial hierarchies, through everyday interactions and institutional practices (Caffrey, 2025; Lee et al., 2017). The term “Asian American,” coined in 1968 by Asian American activists, was intended as a unifying label meant to represent collective resistance to systemic barriers (Lee, 2015; Yoo et al., 2021). As such, Asian American racial identity reflects a shared sociopolitical history shaped by the racialized experiences of diverse, multiethnic, and transnational Asian communities (Yoo et al., 2021).

Despite these political origins, Asian Americans are often subject to homogenizing assumptions about their cultural traits or abilities (Au, 2022; Lee et al., 2017). For example, Asian American adolescents often learn, interpret, and negotiate the messaging of the model minority stereotype, which portrays Asian Americans as inherently high achieving while ignoring the structural barriers and individual struggles they may face (Au, 2022). Asian American adolescents may also encounter racialized expectations that position them as perpetual foreigners, such as being questioned about their national origin or treated as less authentically “American” than their peers (Lee et al., 2017; Woo et al., 2020). In a broader sociopolitical context, Asian Americans are often perceived as being apolitical despite the long history of activism within this pan‐ethnic community and voting barriers this group continues to face (Wong & Ramakrishnan, 2023; Wray‐Lake et al., 2017). Together, these experiences contribute to the development of one's racial identity. Given that racialization is a central experience for Asian Americans in the United States, scholars rooted in Asian Critical Theory emphasize the importance of understanding how Asian Americans develop both their racial and ethnic identities (Juang et al., 2017; Maeda, 2009; Yoo et al., 2021).

Building on this conceptualization, Yoo et al. (2021) introduced AARIIV, which captures one dimension of Asian American identity: the endorsement of specific ideological values tied to this collective experience of racialization. Importantly, AARIIV does not indicate the extent to which individuals are or perceive themselves to be “Asian American.” Rather, it reflects three ideological values: (a) Asian American unity, (b) interracial solidarity, and (c) transnational critical consciousness (Saavedra & Yoo, 2023; Yoo et al., 2021). Asian American unity reflects the value of a shared sense of struggle and solidarity across intersecting identities (e.g., race, gender, class) and diverse backgrounds (e.g., ethnicity, nativity). Interracial solidarity is the individual value that emphasizes the importance of building coalitions between Asian Americans and other racial groups to resist systemic oppression. Transnational critical consciousness captures the value of having the awareness of global systems of racism and imperialism and the exploitation of Asians worldwide. Developing these ideological values is particularly important for fostering critical consciousness and collective action among Asian Americans (Yoo et al., 2021).

Although related, Asian American identity and AARIIV represent two distinct but complementary constructs. Asian American identity refers to individuals' broader understanding of and sense of belonging to the Asian American racialized group, aligning with conceptualizations of the meta‐construct ethnic–racial identity that includes the beliefs and attitudes that individuals have about their ethnicity and race (Umaña‐Taylor et al., 2014). In contrast, AARIIV reflects the extent to which individuals endorse specific ideological values (i.e., Asian American unity, interracial solidarity, and transnational critical consciousness) tied to the collective Asian American identity (Yoo et al., 2021). AARIIV is not intended to capture the overall strength or importance of one's Asian American identity nor does it assess identification with ethnic cultural practices or traditions. Hence, individuals may feel a strong sense of belonging to the Asian American community, highlighted through Asian American identity without necessarily endorsing these ideological values, which is captured by AARIIV. In other words, Asian American identity captures how individuals understand and feel about their membership in the group, whereas AARIIV reflects the extent to which they endorse ideological values tied to that membership.

Emerging studies have begun to empirically examine AARIIV across Asian American college students and adolescents (e.g., Atkin et al., 2023; Saavedra & Yoo, 2023; Yoo et al., 2021). Among college‐aged samples, stronger endorsement of AARIIV has been associated with greater racial centrality, ethnic pride, active engagement with one's ethnic community, and critical consciousness (Yoo et al., 2021). Additionally, endorsement of interracial solidarity and Asian American unity mediated the relationship between critical reflection and collective action, which are domains of critical consciousness (Saavedra & Yoo, 2023). Although most empirical work has focused on college‐aged samples, emerging evidence suggests that these values are also observable during adolescence. For instance, maternal ethnic–racial socialization strategies that promote both cultural heritage and engagement with mainstream society have been linked to higher endorsement of AARIIV among Asian American adolescents (Atkin et al., 2023). Given that most empirical studies have been conducted with college‐aged populations, the present study focuses on college students while drawing on their reflections of high school experiences. Our approach aligns with Yoo et al.'s (2021) call to examine the ecological contexts that inform the development of AARIIV and reflects a developmental perspective in which adolescence represents a key developmental period for exposure to and engagement with these values, even as they may be more readily articulated in early adulthood (Umaña‐Taylor et al., 2014; Yoo et al., 2021).

### Organized after‐school activities as supportive structures for Asian American adolescent development

In a recent paper, we bridged theories on organized activities and ethnic–racial identity development (Eccles & Gootman, 2002; Simpkins et al., 2017; Umaña‐Taylor et al., 2014) to propose a framework outlining how organized activities may support adolescents' ethnic–racial identity development (Kiyama et al., 2026). This framework suggests that organized activities can function as important structural supports for adolescents' ethnic–racial identity development through three interrelated mechanisms: (1) ethnic–racial socialization practices embedded in culturally responsive activity quality features, (2) exposure to same‐ethnic–racial peers and staff at the activity, and (3) the presence of a thriving activity environment that provides the supportive conditions for adolescents to feel comfortable to engage in ethnic–racial identity promoting processes (Kiyama et al., 2026; Figure S1).

The first mechanism involves ethnic–racial socialization practices within organized activities (Kiyama et al., 2026). Through this mechanism, organized activities can directly promote adolescents' ethnic–racial identity development by providing opportunities for skills building (e.g., offering lessons to learn the language of adolescents' cultural heritage), promoting efficacy and mattering (e.g., discussions on resisting negative narratives and build a positive image of their ethnic–racial groups), fostering a sense of belonging (e.g., host events related to adolescents' cultural heritage), and integrating family and community (e.g., activity curriculum is built with adolescents' parents and honors their cultural values) (Ettekal et al., 2020; Kiyama et al., 2026; Umaña‐Taylor et al., 2014). For example, organized activities can help transmit cultural values, traditions, and pride, while also facilitating conversations on discrimination and structural inequities through celebrations, mentorship, and skill‐building opportunities (Hughes et al., 2006; Huguley et al., 2019; Umaña‐Taylor & Hill, 2020). For Asian American adolescents, programs with an explicit ethnic–racial focus have been particularly effective in supporting identity development and critical engagement with racial identity (Chang & Shih, 2021; Kiyama et al., 2026; Kwon, 2013; Tran & Bifuh‐Ambe, 2021; Wu et al., 2019).

A second key direct mechanism involves exposure to same‐ethnic–racial peers and staff at organized activities, who serve as critical socializing agents (Kiyama et al., 2026). Although ethnic–racial socialization research has historically focused on parents as primary socializing agents, recent scholarship emphasizes that adolescents also receive ethnic–racial messages through peers and community contexts, including organized after‐school activities (Ruck et al., 2021; Sladek et al., 2022). Within these contexts, peers represent an important source of ethnic–racial socialization, as adolescents frequently discuss shared cultural experiences, interpret racialized events, and make meaning of their identity‐related experiences together (Wang & Lin, 2023). Within the school literature, the ethnic and racial composition of schools and friendship groups has been shown to shape how adolescents perceive and engage with their ethnic–racial identities (e.g., Phinney et al., 2001; Yip et al., 2013). For instance, adolescents with a higher proportion of same‐ethnic friends tend to explore their ethnic identity more (Kiang et al., 2010) and feel stronger centrality (Kiang & Fuligni, 2009) compared to those with more diverse friendship groups. Similarly, the amount of contact adolescents have with same‐ethnic peers has been linked to stronger feelings of ethnic–racial identity affirmation, belonging, and exploration (Phinney et al., 2001). Interacting with individuals from the same ethnic or racial background facilitates conversations about cultural heritage, engagement in cultural practices, and provides support for identity‐related stressors (Golden et al., 2022; Kao & Joyner, 2004; Syed & Juan, 2012). Peer interactions are particularly important for fostering ethnic identity among Asian American adolescents (e.g., Huang, 1998; Kiang et al., 2007). Moreover, experiences of discrimination can heighten adolescents' awareness of structural racism and strengthen racial identification (Duncan, 2012; Kim, 2013; Tran & Curtin, 2017). Since adolescents are more likely to share stories of racial and ethnic discrimination with their peers than with parents (Syed, 2012), peer interactions—particularly with same‐race and same‐ethnic peers—may serve as a key process for fostering AARIIV. Additionally, having activity staff and peers who share the same ethnic–racial backgrounds enhances the relevance of program content and provides psychological support through shared cultural understanding (Ngo et al., 2018; Wu et al., 2019).

The third mechanism involves the presence of a thriving activity environment that offers warmth, support, and psychological safety (Kiyama et al., 2026). Although these activity features may not directly provide identity‐promoting processes as the two mechanisms described above, they create the relational and emotional conditions that allow adolescents to engage with those processes. In this framework, a thriving activity environment refers to settings characterized by physical and psychological safety, supportive relationships with activity staff and peers, culturally responsive appropriate structure, and positive social norms that affirm adolescents' identities (Eccles & Gootman, 2002; Kiyama et al., 2026). The importance of a thriving activity environment is based on the literature underscoring that adolescents are more receptive to ethnic–racial socialization messages when they experience positive relationships with staff and peers and feel respected and valued within the activity environment (Kulish et al., 2019; Umaña‐Taylor & Hill, 2020).

Conversely, the absence of a supportive environment can undermine identity development and increase feelings of marginalization (Lin et al., 2016; Ma et al., 2020; Priest et al., 2014; Rogers et al., 2020; Umaña‐Taylor & Hill, 2020). In such contexts, adolescents may experience identity conflict or engage in processes of resistance and renegotiation rather than affirmation of their ethnic–racial identities (Umaña‐Taylor et al., 2014). More broadly, although this framework focuses on the promotive ways that organized activities can support adolescents' ethnic–racial identity development, it is important to acknowledge that activities may also involve null or negative processes. As evident with the studies mentioned above, adolescents may encounter racialized experiences within activities such as exclusion or microaggressions (Lin et al., 2016) that can constrain or complicate identity development. Racialized experiences within activities may limit opportunities for exploration and affirmation or prompt adolescents to engage in resistance or renegotiation of their identities. Thus, organized activities should not be assumed to uniformly promote positive developmental processes; rather, the positive impact depends on the extent to which they provide supportive, inclusive, and culturally responsive thriving environment. In this way, a thriving activity environment can be understood as a foundational condition that supports adolescents' engagement with ethnic–racial identity‐promoting processes.

Importantly, beyond these negative experiences, activities may also involve null processes in which ethnic–racial identity is not meaningfully engaged. The framework suggests that the ways activities support ethnic–racial identity development may differ depending on whether activities explicitly center their program content around adolescents' ethnic–racial background. In activities that explicitly center adolescents' cultural heritage, identity‐promoting processes such as ethnic–racial socialization are embedded directly within program design and curricula. In contrast, in more general activities (e.g., math club or sports), ethnic–racial identity development may occur more indirectly through relational and contextual mechanisms such as a thriving activity environment (Kiyama et al., 2026). In the absence of these conditions, ethnic–racial processes may be minimal or not engaged at all. In such cases, adolescents may not perceive their participation as relevant to their ethnic–racial identity development, resulting in null or limited impact of organized activities on identity‐related outcomes.

Although community‐based organized activities have long been recognized for their positive impact on Asian American adolescents' positive development (Chang & Shih, 2021), it is important to acknowledge that Asian American college students participate in a variety of activities besides community‐based activities whose focus may not be on their ethnicity or race (e.g., sports). Community‐based organized activities may have been the focus of studies on this topic because they often incorporate culturally relevant content (e.g., traditional arts, language, and history) and foster a supportive peer environment where adolescents feel comfortable discussing issues related to race and culture (Kwon, 2013; Ngo et al., 2018; Paik et al., 2017; Reyes, 2017; Trieu & Lee, 2018). In a recent systematic review that examined the relations between the processes within organized after‐school activities and adolescents' ethnic–racial identity development, the authors note that the majority of existing empirical studies on this topic examined activities with programmatic goals centered on race or ethnicity or activities that provided opportunities for adolescents to learn about their ethnic–racial heritage even if not explicitly centered on race or ethnicity (Kiyama et al., 2026). Hence, it remains unclear whether these ethnic–racial identity‐promoting processes within activities operate similarly across a broad array of organized activities that do not explicitly focus their programmatic content on race or ethnicity. Moreover, although prior research has largely emphasized promotive processes, literature also suggests that organized activities can also involve negative or constraining experiences such as microaggressions that may inhibit adolescents' ethnic–racial identity development (e.g., Ettekal et al., 2020; Lin et al., 2016). Hence, addressing this gap is critical for clarifying whether ethnic–racial identity development within activities is primarily driven by the intentional design of activities centered on race or ethnicity or whether identity‐promoting processes may also emerge through more general activity contexts that provide supportive and relationally rich environments as well as under what conditions give rise to negative ethnic–racial identity processes. Considering a range of organized activities allows for a more nuanced understanding of which different activity contexts support Asian American adolescents' ethnic–racial identity development. Furthermore, understanding how organized activities contribute to Asian American adolescents' racial identity ideological values—even when activities lack an explicit ethnic–racial focus—can provide insights into how activities can create supportive developmental environments.

## CURRENT STUDY

What does it mean to be Asian American? As all Asian Americans undergo experiences of racialization in the United States, this question is integral to understanding Asian Americans' ethnic–racial identity development. Given the research gaps mentioned above, the current study qualitatively explored how Asian American college students thought their participation in organized activities during high school related to their Asian American identity and AARIIV. A qualitative approach was particularly well suited to this study given that the goal was not simply to assess whether organized activities are associated with Asian American identity and AARIIV, but rather to understand the nuanced mechanisms and meaning‐making processes through which Asian American college students perceived these activities shaped their identities. Semi‐structured interviews allowed students to reflect on these complex experiences in their own words that may not have been captured through predetermined survey items.

We focused on a college‐aged sample given individuals are better able to reflect and articulate their ethnic–racial identity‐related experiences and beliefs during emerging adulthood (Umaña‐Taylor et al., 2014). At the same time, asking college students to reflect on their high school experiences allows for a retrospective examination of how earlier developmental contexts may have contributed to the formation of these values and beliefs related to their identities (Yoo et al., 2021). We examined how Asian American college students thought their participation in these activities helped or hindered their exploration of Asian American identity and AARIIV. The present study addresses the following research questions: (1) How do Asian American college students believe organized afterschool activities contributed to how they think about their identity as Asian American? and (2) how do Asian American college students think these activities specifically supported their exploration of AARIIV?

## METHOD

### Research design and participants

The present study utilized purposive sampling to recruit Asian American undergraduate students attending a large public university in Southern California that is an Asian American and Native American Pacific Islander‐Serving Institution. As of the Fall 2024 quarter, Asian students were the largest racial–ethnic group (47%) among the undergraduate population at this institution (University of California, 2025). Students who met the following criteria were recruited: (a) identified as an Asian American based on the definition by the U.S. Census Bureau (2020): “A person having origins in any of the original peoples of the Far East, Southeast Asia, or the Indian subcontinent (e.g., Cambodia, China, India, Japan, Korea, Malaysia, Pakistan, the Philippine Islands, Thailand, and Vietnam),” (b) were not an international student, (c) attended high school in the United States for at least 3 years, and (d) participated in at least one organized activity during high school in the United States.

Data collection involved semistructured interviews with 25 Asian American college students (80% female; 96% U.S.‐born) who were in their first to fourth year of college. Eight students identified as Chinese, six as Vietnamese, five as Korean, four as Filipino, three as Japanese, two as Taiwanese, and one as Indian. Among these students, five of them also racially identified as White, given their mixed heritage. In high school, the students participated in a diverse range of organized activities: sports (15%), arts (27%), academic (15%), religious (5%), cultural (8%), volunteer and services (15%), and leadership (14%); 26% of these activities were community‐based. Students participated in an average of 3.12 activities (range: one to seven activities per student). Table 1 provides an overview of the students and their activities.

**TABLE 1:** Demographic Characteristics of Asian American College Students.

Pseudonym (gender^a^)	Race^b^	Ethnicity^b^	Year in college	Organized activities during high school^c^
Emily (F)	Asian American	Chinese American	1	Badminton* Internship Chinese cultural club* Yearbook
Sophia (F)	Asian	Chinese	4	Key club* National Dance Arts Honor Society
Ethan (M)	Asian	Taiwanese	3	Bands (Marching band, a jazz band and a wind ensemble) Mindfulness club* ‐ focused on sharing mindfulness techniques Club Econ Club General – life skills club Boy Scouts* Hospital volunteer Taiwanese club*
Isabella (F)	Asian	Vietnamese	2	AVID program (Advanced Via Independent Determination). ‐ college prep program NJROTC* (Navy Junior Reserve Officer Training Corps) Basketball Key club
Mia (F)	Mixed (Asian and White)	Taiwanese and White	3	Glee club Color guard* Musical theatre Church youth group Reading Partners ‐ a tutoring program to help elementary school kids with their reading. Girl Scouts
Hannah (F)	Asian	Vietnamese	2	Track and field*
Jason (M)	Asian	Japanese	1	Boy Scouts* Coastline Regional Occupational Program ‐ career oriented program Taiko* – a Japanese traditional instrument club Church youth group*
Lily (F)	Mixed (Asian and White)	Japanese/white (dad) + Vietnamese/Indian (mom)	2	Tennis*
Grace (F)	Asian	Vietnamese	4	Dance Tennis Key club* UNICEF volunteer APIC* (Asian Pacific Islander Club)
Ava (F)	Asian	Vietnamese	3	Museum‐based art club
Zoe (F)	Asian	Chinese	4	Button club ‐ a club making pin back buttons. Pokémon club*
Olivia (F)	Mixed (Asian and White)	Filipino and European	2	Student government*
Natalie (F)	Asian	Filipino	2	Orchestra* ‐ community‐based program
Lauren (F)	Asian American	Filipino Chinese	2	Robotics Band Drama Church youth group*
Jessica (F)	Asian	Korean	3	Student government* National Honor Society Cheer
Ashley (F)	Asian American	Korean and White	2	Creative writing*
Victoria (F)	Asian	Chinese	3	Soccer* Volleyball Student relational club Church youth group
Chloe (F)	Asian	Korean	4	Early College Program Swim National Honor Society Girl Scouts
Samantha (F)	Chinese American	Chinese	1	Dance* Link Crew Chinese folk dance*
Rachel (F)	Asian	Filipino	1	Music program* Volunteer club Badminton
Ryan (M)	AAPI	Korean	3	Photography Key club Political campaign volunteer* Research Debate
Evelyn (F)	Asian (East)	Chinese, Japanese	3	Dance* Cheerleading
Madeline (F)	Asian	Korean	4	Badminton* Tutoring Key club Culture club* Academic Decathlon
Daniel (M)	Mixed (Asian and White)	Chinese	1	Youth civic council* Tutoring Tennis
Kevin (M)	Asian	Vietnamese	4	Swim Pep band

*Note*: Besides Evelyn, who came to the U.S. at 14 months old, all participants were U.S. born.

^a^ F = female, M = male.

^b^ Race and ethnicity were asked as open‐ended questions.

^c^ Activities with an asterisk were associated with Asian American identity and AARIIV.

Recruitment and data collection proceeded iteratively, and thematic saturation was reached after 25 interviews, that is, when additional interviews did not yield substantially new themes or perspectives relevant to the research questions (Guest et al., 2006). Furthermore, as mentioned above, the final sample included students with experiences in diverse activity types. Thus, we determined that the final sample size was sufficient to support in‐depth qualitative analysis of Asian American college students' experiences.

### Interview procedures

Semistructured qualitative interviews with the 25 Asian American college students were conducted in the spring and summer of 2024. The interviews generally lasted between 45 and 90 min. Interviewers, including the first author and research assistants, were trained in research ethics and effective interviewing techniques. During data collection, weekly meetings were held with the research team to discuss and debrief any issues that arose from the interviews. Students were interviewed in English, and the audiotaped interviews were transcribed verbatim. Pseudonyms were used in all of the interview data. Each student provided consent and received a $10 honorarium for participating in the interviews.

The semistructured interview script contained multiple open‐ended questions that were developed based on existing literature (e.g., Simpkins et al., 2017; Yoo et al., 2021) and conversations with the research team. The first section of the interview included general questions about students' experiences being Asian American during high school (e.g., “How did you feel about the term ‘Asian American’ in high school?”). The second section of the interview focused on questions about the students' participation in organized activities during high school, asking about their motivations, experiences, and role these activities played in shaping their understanding of their racial and ethnic identity (e.g., “Can you share your overall experience participating in organized after‐school activities in high school?”). The section of the interview included questions to address the first research question (e.g., “Could you describe the organized after‐school activities that made an impact on your understanding of being Asian American?”) and the second research question (e.g., “Did your organized afterschool activity provide opportunities to talk about your race and other racial groups in the U.S.?”). When responding to questions about their exploration of AARIIV, students were asked to focus on one activity that had a positive impact on their understanding of being Asian American as a way to anchor their reflections to one salient activity context and to understand what activities may or may not have been doing well in supporting Asian American identity development. The subsequent questions about AARIIV were exploratory and did not presuppose that these experiences were exclusively affirming. This approach allowed participants to provide deeper reflection on identity‐related processes within a specific activity context rather than offering brief accounts across multiple activities. Interview questions were intentionally structured to begin with broad questions about students' overall activity experiences and their understanding of being Asian American before asking them to identify one activity that had the most meaningful impact. This sequencing encouraged students to reflect across the full range of their activities and select a context that was most salient for their identity development. Focusing on one activity builds on the approach often taken by narrative identity approach, which argues that individuals make meaning of their identity by examination of salient or self‐defining experiences (McAdams, 2001). This approach also aligns with prior research on organized activities that has examined adolescents' experiences within a single target activity to better understand developmental processes and features of high‐quality activity settings (e.g., Hansen et al., 2003; Larson et al., 2006). In addition, the questions assessing AARIIV were designed to probe more specific ideological values (i.e., Asian American unity, interracial solidarity, and transnational critical consciousness), moving beyond general reflections on Asian American identity to capture how participants engaged with these distinct values related to Asian American racial identity. Expanding on this, the final section of the interview included questions pertaining to how their organized activity may have helped explore their AARIIV (e.g., “Did [name of organized activity] provide opportunities for interracial solidarity and learning about the struggles of other communities of color [non‐White communities such as African, African American, Asian, Latino, Middle Eastern and North African, Native American, Pacific Islander, and Slavic]?”). In the interviews, questions about Asian American identity focused on how students understood and reflected on being Asian American as a broader ethnic–racial identity construct, whereas questions about AARIIV specifically addressed the three ideological values associated with Asian American racial identity (i.e., Asian American unity, interracial solidarity, and transnational critical consciousness). A complete list of the interview questions can be found in Appendix A.

In addition to the semi‐structured interviews, students' demographic information (e.g., gender, nativity status, and parents' highest level of education) was collected via a post‐interview survey (see Appendix B).

### Coding and analysis

The interview data were qualitatively coded through multiple stages using both inductive and deductive coding approaches to identify patterns and themes in the data (Saldaña, 2016). To assist in data analysis, features in the qualitative coding software called Dedoose Version 9.0.107 and Microsoft Excel spreadsheets were used.

In the first stage of coding, the first author read through a subsample of interviews (i.e., 8 of 25 interviews) in their entirety and placed particular attention on responses related to the research questions. Inductive and deductive approaches were used where codes were developed from both the data and prior literature using in vivo (i.e., verbatim words or phrases from participants) and descriptive (i.e., words or phrases that summarize a topic based on prior literature) coding techniques (Saldaña, 2016). During the inductive coding phase, the first author identified preliminary codes directly from the Asian American college students' responses, attending to patterns and meanings reflected in their language and perspectives (e.g., students describing activities as spaces where they could “learn about their cultural heritage,” “feel a sense of belonging,” or “have a trusting relationship with activity staff or peers.”). For the deductive phase, the framework on organized activities and ethnic–racial identity development (Kiyama et al., 2026) was used to refine the codes by incorporating theoretically informed categories such as ethnic–racial socialization within activities, AARIIV, and features of a thriving activity environment. For example, the inductive of code of “learning about their cultural heritage” was later grouped within the broader category of ethnic–racial socialization during the development of the coding framework. The first stage of coding yielded a preliminary coding framework that largely had two foci: (a) how Asian American college students thought their organized activities during high school helped them understand their Asian American identity and (b) how Asian American college students thought their organized activities supported their exploration of AARIIV.

In the second stage of coding, the first author applied the preliminary coding framework to all 25 participant interviews. The coding framework was revised based on coding the remaining data, reviewing written memos of the coding process, previous literature, and discussions with the research team. The purpose of this stage was to create a stronger coding framework that captured the unique perspectives of all Asian American college students in the study. To assess reliability of the finalized coding framework, the first author trained two research assistants using the finalized coding framework and reviewed sample excerpts together to ensure shared understanding of the coding framework. The two trained research assistants then independently coded 20% of excerpts drawn from all 25 interviews. Inter‐rater reliability between the first author and research assistants indicated strong agreement, with an average Cohen's kappa coefficient of *k* = .88.

In the third and final stage of coding, broader themes were created by the first author by consulting the coding framework, interview data, previous literature, and written memos. Developed themes were also presented to the members of the research lab, which included a team of doctoral students and the second author, and refined based on their feedback. The themes were based on the common patterns we found across the codes from the previous coding stage. The goal was to create themes that best captured the connections between how participants thought their experiences in organized activities impacted their understanding of being Asian American and what aspects of their activities they perceived as helpful in becoming aware of their AARIIV (Ryan & Bernard, 2003).

### Positionality

Acknowledging our positionality and reflecting on how our experiences shape our approach to the present study is important. The first author identifies as Asian and Japanese, with a bicultural background from growing up in both the United States and Japan, which strongly informs her interest in how community‐based organized after‐school programs provide structural support for Asian American youths' positive development. Additionally, her experience as an instructor in out‐of‐school settings, particularly those serving Asian and Asian American youth, further influences her approach to the current data. The second co‐author is a White, female raised in California whose family has been in the United States for more than three generations; her expertise is in developmental psychology and focuses on youth's organized afterschool activities, including the predictors of participation, developmental processes, the outcomes associated with participation, and examining diversity in activities.

## RESULTS

### Activities associated with students' understanding of being Asian American

To address the first research question—How do Asian American college students believe organized afterschool activities contributed to how they think about their identity as Asian American?—we examined students' reflections on their participation in a wide range of activities (Table 1). Although students' motivations for participation often included practical benefits, such as beneficial for college applications and meeting parental expectations, many described these activities played a deeper role in shaping their understanding of being Asian American, which is a core dimension of one's ethnic–racial identity (Umaña‐Taylor et al., 2014). Consistent with the meta‐construct of ethnic–racial identity (Umaña‐Taylor et al., 2014), students' reflections primarily reflected processes of affirmation (e.g., feelings of connection and belonging) and exploration (e.g., reflection and learning about their identity).

For most students, though they did not necessarily join activities with the goal of exploring their racial or ethnic identity, they described having opportunities to explore their identities as Asian Americans through participation and relationships formed within these settings. Of 25 students, 21 felt specific activities helped them develop a deeper sense of their Asian American identity. The activities included sports (*n* = 4, e.g., badminton), arts (*n* = 8, e.g., color guard), religious (*n* = 2, e.g., church youth group), cultural (*n* = 6, e.g., Chinese cultural club), volunteer and service (*n* = 4, e.g., key club), and leadership (*n* = 5, e.g., Boy Scouts), with eight (28%) of them being community‐based organized activities (see Figure S2). Aligned with the framework shown in Figure S1 (Kiyama et al., 2026), students' reflections pointed to two interrelated ways in which they thought organized activities shaped their understanding of being Asian American (i.e., their broader ethnic–racial identity): (1) exposure to same or different ethnic–racial peers and activity staff and (2) ethnic–racial socialization through inclusion and learning of Asian American histories and cultural practices. The two mechanisms are discussed next and presented in Table 2.

**TABLE 2:** Organized after‐school activities associated with students' understanding of being Asian American.

Theme	Sub‐theme	Type of activities	Example quotes
Exposure to same or different ethnic–racial peers and activity staff (*n* = 21)	Exposure to Asian or Asian American peers and activity staff (*n* = 19)	Sports (*n* = 1; badminton) Arts (*n* = 6; e.g., color guard) Religious (*n* = 1; church youth group) Cultural (*n* = 3; e.g., Chinese cultural club) Volunteer and services (*n* = 2; e.g., key club) Leadership (*n* = 6; e.g., Boy Scouts)	“I feel that dance is not really something I feel like Asians would really do, but joining dance made me find people who are Asian American. … I'm glad that I was able to find other people who were like me and that wanted to do the same thing as me.” (Samantha, Dance) “It was [predominantly] Southeast Asian and parents were very involved in the program. … just being around more adults in my own community was able to open up my perspective a little bit more. … being around more adults that were open to teaching and open to talking, they kind of brought more understanding of my own communities to the program.” (Isabella, NJROTC)
	Exposure to predominantly non‐Asian peers and activity staff (*n* = 3)	Sports (*n* = 2; e.g., track and field) Religious (*n* = 1; church youth group)	“All the kids were all White, and then there were only some people of color, and then very few Asians. So we stood out like a sore thumb. And Asians aren't usually fast. … my coach didn't see my potential until season was about to start. And he put me on varsity. … I wasn't ashamed to be an Asian American, but I guess it just made me more aware of the social setting I was in. … made me realize I may look different from everyone else, I might not fit in with them culturally, but I'm here now and it made me more adaptable and more open in working with other groups no matter what the cultural difference was.” (Hannah, track and field) “You're sort of always reminded of the fact that there are ethnic differences or sociocultural differences … The Asian kids who played tennis also were expected to be doing well in school … people could say you're Asian American, you're going to be more obedient and you're going to be more coachable, that sort of thing.” (Lily, tennis)
Ethnic–racial socialization (*n* = 17)	Inclusion of Asian cultural celebrations (*n* = 9)	Arts (*n* = 3; e.g., music program) Volunteer and services (*n* = 2; e.g., key club) Leadership (*n* = 4; e.g., student government)	“Because we had such a large Asian population at our school, we also focused on Asian holidays as well, such as Lunar New Year… I also was required to research up on these holidays and Asian culture so we could accurately represent it and celebrate it correctly and celebrate it with the most respect that we could. So I feel like that really helped impacted me.” (Jessica, student government) “When we sing, we don't just sing straight English songs, we diversify. We've probably sung a few songs from Africa, few Spanish songs, few Korean songs. But they did do one song that was specifically Filipino. So that was great. I got some representation there. … It was great. I mean, it was a song that I grew up with, so it was really nice to see.” (Natalie, orchestra)
	Engagement with Asian American history and cultural practices (*n* = 8)	Arts (*n* = 2; e.g., Chinese folk dance) Religious (*n* = 1; e.g., church youth group) Cultural (*n* = 3; e.g., Asian Pacific Islander club) Leadership (*n* = 2; youth civic council)	“We learned and made presentations about it [different countries]. … So I got to learn a lot more about not just Asian countries, but all the other ones too.” (Madeline, culture club) “I'm kind of seeing how much work oriented and how overworked a lot of people get in our community. … one of the activities we had was opening up about how you feel about work and stuff … it is good to set yourself high standards to become better than you were yesterday. … we definitely noticed that a lot of Asian parents do push the idea of working really hard and it is tough on a lot of us.” (Ethan, mindfulness club)

*Note*: Participants were allowed to answer any number of activities that they thought foster their understanding of being Asian American. Sample size indicated as “*n*” is the number of participants.

#### Exposure to same or different ethnic–racial peers and activity staff

In relation to students' broader understanding of their Asian American identity, this mechanism operated through everyday interactions and shared experiences with peers and activity staff. Nineteen students highlighted that they thought the presence of Asian or Asian American peers and staff in their activities helped foster a sense of connection to their Asian American identity, reflecting ethnic–racial identity affirmation (Umaña‐Taylor et al., 2014). This theme emerged across various activity types with arts, cultural, and leadership activities being the most frequently mentioned. Mia, who participated in color guard, described the subtle but powerful comfort of being surrounded by people who shared her background:

There were just so many other Asian Americans and also other Wasians [White Asians]. So just being able to be in that space with them was really important for being able to shape who I am. … We weren't sitting around discussing what it means to be Asian, but we were just hanging out together. And up until that point, I hadn't really done that with non‐white people.

Similarly, Rachel, who was involved in a music program, reflected on the emotional safety of being part of an Asian‐majority space:

I felt like I wasn't different or excluded and I felt like it was a safe environment for me because I had people who were going through the same things as me or have experienced the same things as me, enjoy the same things as me.

Students' descriptions of comfort, belonging, and positive regard reflect ethnic–racial identity affirmation towards their Asian American identity. Even for students who grew up in predominantly Asian communities, these peer networks within activities reinforced a sense of shared identity and understanding. The comfort of not having to explain oneself or stand out allowed students to connect more deeply with their ethnic–racial identity.

Although exposure to same‐ethnic–racial peers and activity staff was often described as providing comfort, three students noted that participating in activities with predominantly non‐Asian peers also heightened their awareness of being Asian American. Students expressed feeling different or sometimes experiencing microaggressions, which prompted reflection on their Asian American identity, consistent with processes of ethnic–racial identity exploration (Umaña‐Taylor et al., 2014). Hannah, a track and field athlete, recalled experiencing microaggressions and lowered expectations based on her race: “made me more aware that while I do look different, I wasn't ashamed to be Asian American.” These experiences, although sometimes uncomfortable, were described as prompting reflection on their ethnic–racial identity and how others perceived them.

#### Ethnic–racial socialization—Inclusion and learning of Asian American histories and cultural practices

Some students described experiencing ethnic–racial socialization in organized activities through the inclusion of Asian American cultural celebrations and opportunities to learn about Asian American histories and cultural practices. In relation to their broader ethnic–racial identity, nine students recalled that they thought the inclusion of cultural celebrations within their activities—whether formally organized or informally encouraged—played a meaningful role in shaping their understanding of being Asian American. Participants often mentioned leadership‐focused (e.g., student government) or service‐oriented (e.g., key club) activities. Jason's Boy Scout's troop, for example, incorporated Japanese traditions and Buddhist practices, even though the organization itself is rooted in Christian values:

Because we're so heavily invested in our Japanese side. It attracted other new members from outside the troop … For example, there was a lot of food, a lot of aspects like onigiri and then for our celebrations it'd be very Japanese, we'd go to a sushi bar or we'd go to some Ramen place.

Students described these cultural markers as helping them feel seen and validated, reflecting ethnic–racial identity affirmation, and blending American and Asian identities in ways that felt meaningful and reflecting a more coherent and integrated understanding of their ethnic–racial identity (Umaña‐Taylor et al., 2014).

Eight students highlighted activities that centered on teaching Asian American history and cultural practices. The types of activities were predominantly leadership (e.g., youth civic council) and cultural activities (e.g., Taiwanese club). Ethan, who was involved in the Taiwanese club, reflected on how the club's events and programming expanded his appreciation for his ethnic background:

We definitely spent a lot of time working with guest speakers as well as demonstrations and lectures and performances on things about our culture. … I learned a lot more about Taiwan and our appreciation more than I would have. … they had us volunteer and do a performance for a Taiwanese festival as well as helping the volunteer to run booths and get to know Taiwanese events around the community.

Similarly, Grace shared how participating in the Asian Pacific Islander Club gave her the opportunity to learn “more than just my ethnic background” and learn more about other Asian ethnic groups in the United States, which gave her clarity about the meaning of group membership (Umaña‐Taylor et al., 2014).

Taken together, these experiences illustrate how students described organized activities as offering ethnic–racial socialization through cultural celebrations and teaching histories and cultural practices of Asian American cultures into the activity content. Whether through food, traditions, or engagement with Asian American histories, students perceived these activities provided meaningful contexts for connecting with their heritage while also fostering a broader understanding of Asian American identity.

### How students thought organized after‐school activities supported their AARIIV

Whereas the previous section focuses on students' reflections on their broader ethnic–racial identity (i.e., how they understand and feel about being Asian American), this section highlights how students described their activities as supporting engagement with AARIIV, that is, the endorsement of the three ideological values including Asian American unity (i.e., shared sense of struggle and solidarity across intersecting identities and diverse backgrounds), interracial solidarity (i.e., the importance of building coalitions between Asian Americans and other racial groups) and transnational critical consciousnesses (i.e., having the awareness of global systems of racism and imperialism and the exploitation of Asians worldwide) (Yoo et al., 2021). To address the second research question—How do Asian American college students think these activities specifically supported their exploration of AARIIV?—we asked students to reflect on whether their activities facilitated engagement with these values. Sixteen students identified activities they felt supported engagement with AARIIV, including sports (*n* = 4, e.g., track and field), arts (*n* = 4, e.g., Orchestra), cultural (*n* = 2, e.g., Asian Pacific Islander Club), volunteer and service (*n* = 2, e.g., youth civic council), and leadership (*n* = 4, e.g., Boy Scouts). Of these, five (31%) were community‐based organized activities. Details are presented in Table 3.

**TABLE 3:** Mechanisms within organized after‐school activities that supported Asian American racial identity ideological values.

Theme	Subtheme	Type of activities	Example quotes
Exposure to same or diverse ethnic–racial peers (*n* = 10)	Peer dynamics (*n* = 4)	Sports (*n* = 2; e.g., tennis) Arts (*n* = 1; music program) Volunteer and services (*n* = 1; political campaigning)	“I think it [track and field] does serve as a great platform because it just attracts all different types of races and it really doesn't matter where you're coming from as long as you're putting in the hard work and results are showing it seriously does not matter. That's the beauty of it. … realize we people who are just not Caucasian, we're all riding in the same boat because you just have the same kind of patterns. … being surrounded by different people, different mindsets. It is truly enrichening.” (Hannah, track and field) “It is a competitive environment, and I would say there is existing racial tensions that are perpetuated in the sports environment. That informed my more, I guess, accepting attitude that I have today. … I would have friends who are Asian, who lived in relatively impoverished conditions, but showed up to the same court as me and played tennis.” (Lily, tennis)
	Peer discussions (*n* = 6)	Sports (*n* = 2; e.g., soccer) Arts (*n* = 1; color guard) Cultural (*n* = 1; Pokemon club) Leadership (*n* = 2; e.g., Boy Scouts)	“The biggest thing is that having those informal discussions, people would be able to complain about different struggles or recent issues … that was really helpful in shaping the way that I processed the pandemic Asian hate crimes that were happening and things like that. … it was more focused on issues within the Asian community, but since there was so many different backgrounds within that in our group, I was able to learn more about their different cultures as well and different struggles, which was really helpful because prior to that, I had not really even been able to talk to Chinese people or Taiwanese people. So I was like, oh my gosh, there are so many different backgrounds. And I think through color guard also being able to understand, okay, this is what it means to be Korean and this is what Vietnamese means. I think that was also really helpful.” (Mia, color guard) “Being able to have conversations with other Filipinos … talking around culture and being able to bond with people on that level. … even if I'm not in the Philippines, I can show aspects of that in my life. There were a couple international students in our group, so talking to them and learning about what their life is like back home and what their feelings like back home, their culture. … their struggles from coming overseas into America and to adjust to American culture and not knowing the language as well as others.” (Olivia, student government)
Ethnic–racial socialization (*n* = 7)	Intentional activity content (*n* = 7)	Arts (*n* = 2; e.g., orchestra) Cultural (*n* = 2; e.g., APIC) Volunteer and services (*n* = 1; youth civic council) Leadership (*n* = 2; e.g., student government)	“We were well‐informed of other communities and other countries and cultures because we would have to learn about it for the academic version of our competition. … Because we competed in those competitions, people on those particular teams were able to have a deeper understanding of those cultures and learn more about them.” (Isabella, NJROTC) “We had presentations that allowed us to explore other ethnicities and racial backgrounds of Asian descent. So that really expanded everyone's knowledge of these different groups and yes. … We felt more connected. I would say me and other participants could relate to each other in our identity in how we grew up. … even though we share similar cultural traditions and or experiences at home, there's a broader more, I guess, experience, experiences that I haven't faced but others have. And even though we share a lot of similar things, we're also very different as well.” (Grace, APIC)

*Note*: When reflecting on their exploration of Asian American racial identity ideological values, students were asked if their organized activity/activities positively influenced their understanding of being Asian American. Participants then responded to questions about whether this activity facilitated engagement with the three values of Asian American racial identity ideological values. Sample size indicated as “*n*” is the number of participants.

Similar to the previous section, students described two mechanisms in their activities as supporting engagement with AARIIV: (a) exposure to same or diverse ethnic–racial peers, which shaped students' experiences through peer dynamics and informal discussions, (b) ethnic–racial socialization, particularly through intentional activity content.

#### Exposure to same or diverse ethnic–racial peers—Peer dynamics and discussions

Students felt the ethnic composition and the relationship dynamics between peers within activity settings shaped their engagement with AARIIV, including Asian American unity, interracial solidarity, and transnational critical consciousness. Four students described how they thought their peer groups shaped these experiences. Rachel, who participated in a music program, reflected on how being part of an activity surrounded by Asian American peers of diverse ethnic groups reinforced a sense of unity despite their diversity. She described how “it wasn't always necessarily of our same ethnicity, but we were still kind of united by the fact that we were Asian American and we enjoyed certain types of music.” This reflects Asian American unity, one of the three values of AARIIV that emphasizes solidarity across diverse backgrounds within the panethnic group. Rachel also described how performing and competing with schools that had more racially diverse student populations allowed her to recognize shared experiences across racial groups: “we can connect [with others] because of it.” This reflects interracial solidarity, as she recognized shared experiences and connections beyond her own racial group.

Even when activities did not explicitly provide a space for discussing AARIIV, students felt informal peer discussions—both with Asian American and non‐Asian American peers—created meaningful opportunities for students to reflect and engage with all three dimensions of AARIIV. First, conversations with Asian American peers from different ethnic backgrounds were described as helping students recognize shared experiences, reinforcing a sense of Asian American unity. Second, interactions with non‐Asian American peers were described as encouraging reflection on racial dynamics beyond the Asian American experience, creating opportunities for interracial solidarity. Third, conversations with international Asian students were described as introducing perspectives that extended beyond the U.S. context, supporting awareness of transnational critical consciousness. Victoria who participated in soccer, described how having hard conversations deepened her awareness of both shared and distinct struggles across differing ethnic–racial groups:

There's a lot of things that are rooted in our culture that aren't shown based off of appearances, … being in those relationships made more open‐minded for other people and in other social issues to be able to see them from different perspectives versus only my own.

Students' peer interactions and shared experiences suggest that even when activities were not explicitly focused on AARIIV, these informal exchanges created spaces for students to engage with broader racial and global dynamics consistent with the three values of AARIIV.

#### Ethnic–racial socialization—Intentional engagement with AARIIV

Seven students described how they thought participating in organized activities with structured goals and planned content centered on the three values of AARIIV. For instance, Daniel, who participated in youth civic council, reflected on his advocacy work, which included organizing social justice initiatives such Stop Asian Hate campaigns. He highlighted how the program granted students autonomy in selecting the social issues they wanted to address, fostering a sense of collective responsibility and Asian American unity, which is one of the three dimensions of AARIIV:

They would give us a little bit of guidance in a general direction, and then we would just go about it. We would also vote for which social justice, we would have our group put in, oh, this is what we want for a social justice event.

Natalie, who participated in Orchestra, shared how the activity staff made intentional efforts to create space during the activity to discuss interracial solidarity, especially in the context of 2020 and the Black Lives Matters movement. She noted:

Considering 2020 and BLM, they did pretty good in handling that situation and making sure that we were all heard… instead of having rehearsal, we'd have a meeting or a panel type thing where you were given the chance to just talk about how we felt. … it opens your eyes to see like, oh, this is real. This is not something that's separate, but this is happening to someone that I care about.

Through ethnic–racial socialization, which involved providing intentional activity content engaging with AARIIV, students thought they developed a deeper connection to the Asian American community and fostering solidarity across other ethnic–racial groups.

Our findings underscored that organized activities could provide opportunities for students to engage with AARIIV through peer dynamics, spaces for students to have conversations regarding their racial identity, and activity content intentionally designed to incorporate these values.

### Variability in experiences with AARIIV

Although many students (64%) described organized activities as supporting their exploration of AARIIV, not all students experienced these opportunities. Nine students reported that their activities did not provide opportunities to engage with AARIIV, often responding with statements such as “no” or “I don't think so.” Besides one student who participated in culture club, other students were involved in activities which central focus was not on cultural‐related content: sports (*n* = 1, e.g., swimming), arts (*n* = 5, e.g., dance), volunteer and service (*n* = 1, e.g., key club), and religious (*n* = 1, e.g., church youth group). Students' reflections revealed meaningful variability in how AARIIV was experienced across activity contexts and offered insight into the conditions under which engagement with these values was more or less likely to occur.

Among the nine students who did not perceive their activities as supporting AARIIV, a common theme was limited or constrained engagement with the three ideological values of AARIIV. For some students, this reflected an intentional avoidance of potentially controversial topics. Sophia, for example, described how her activity appeared to avoid engaging in potentially controversial or political discussions: “I think they try their best to not cause controversy.” This suggests institutional boundaries around discussions of race or sociopolitical issues, or discomfort among activity staff in facilitating such conversations. Other students described missed opportunities for intentional socialization. Ava who participated in a museum‐based activity, recalled attending exhibitions centered on Indigenous histories and cultural food traditions but noted that “We surprisingly didn't talk about it enough considering that was the main point of the exhibit.” Similarly, Evelyn expressed that cultural elements such as Asian dance styles or Asian American music could have been incorporated more intentionally into her dance team's performances. Chloe, who participated in swimming, reflected on how some coaches dismissed culturally diverse perspectives, privileging “American customs” over students' varied cultural backgrounds. She explained:

I would see some students who grew up with immigrant parents and they have a different way of doing things, but some of my coaches would completely disregard that and kind of keep, oh, American Customs are the better one. … but everyone has a different upbringing and a different way of just looking at life

Students' reflections suggest that the presence of relevant content or opportunities alone did not necessarily translate into structured dialogue or engagement with the three values of AARIIV.

Another pattern reflected students' uncertainty about whether AARIIV‐related discussions were appropriate or necessary within certain activity contexts. For example, Hannah from track and field explained: “It's a sport at the end of the day and it's not necessarily catering towards connecting yourself to your culture. … I honestly believe it's here for everyone to learn discipline and it doesn't intertwine with the race.” Similarly, Samantha, who participated in pep squad, stated that she “did not feel that there was a need to talk about any ethnicity or racial concerns.” In these cases, students viewed the primary purpose of their activity as unrelated to their racial identity and therefore did not expect such engagement to occur.

### Thriving activity environment of inclusivity and flexible accommodation

In addition to peer interactions and ethnic–racial socializations mentioned in the findings above, students also highlighted the importance of a broader activity environment in shaping how organized activities supported both their understanding of being Asian American (addressed in the first research question) and AARIIV exploration (addressed in the second research question). Specifically, students emphasized that a supportive activity environment characterized by inclusivity, respect, and flexibility created the psychological safety necessary for identity exploration and discussions about race, culture, and social issues.

In this way, a thriving activity environment functioned as an important contextual condition that supported the peer interactions and ethnic–racial socialization processes described above. When students felt that their activity environments were inclusive and responsive to their cultural backgrounds, they reported feeling more comfortable engaging with questions about their Asian American identity and reflecting on broader racial dynamics. Students described two key features of a thriving activity environment: (a) inclusivity and (b) flexible accommodation. Some students emphasized the role of activity staff in promoting inclusivity. Jessica, who participated in student government, described how staff made sure students felt included and welcomed:

The adults in charge actually were not Asian for my student government. … And I think that because of that, they really wanted to make sure that everyone felt included … we were very focused on trying to include multiple cultures.

Students also highlighted how activity staff demonstrated flexibility by accommodating their religious and cultural obligations. For instance, Hannah from track and field shared how staff allowed students to miss practice for religious events like Passover. Similarly, Isabella recalled how NJROTC activity staff made an effort to celebrate cultural traditions, such as Lunar New Year: “during Lunar New Year … they [the non‐Asian activity staff] realized that a lot of students wear it [an áo dài – a traditional Vietnamese garment] and actually celebrate it and they went out and bought their own.” These actions reinforced students' sense of trust and demonstrated the activity staff's investment in their well‐being. Importantly, students explained that this type of supportive environment did not directly teach AARIIV. Rather, it created the relational conditions that allowed students to engage more openly in the peer discussions and ethnic–racial socialization processes through which both Asian American identity exploration and AARIIV engagement occurred.

Findings on a thriving activity environment highlight that even when students did not perceive a direct impact on their AARIIV exploration, they still offered valuable insights into how activities could better engage with them. Greater efforts to integrate diverse perspectives and culturally relevant content may enhance the developmental impact of these activities.

## DISCUSSION

What does it mean to be Asian American? As Asian Americans navigate racialization experiences (e.g., model minority stereotype, perpetual foreigner) in the United States, this question is central to understanding their identity formation (Yoo et al., 2021). To understand how this development occurs within the context of organized after‐school activities, the present study examined how Asian American college students thought their participation in organized activities during high school was related to their understanding of their Asian American identity and engagement with AARIIV. The following research questions were qualitatively explored: (1) How do Asian American college students believe organized activities contribute to how they think about their identity as Asian American? and (2) how do Asian American college students think these activities specifically support their exploration of AARIIV?

### Organized after‐school activity participation and Asian American identity and AARIIV

The present study contributes to the growing body of research on ethnic–racial identity development by highlighting how organized activity participation supports Asian American college students' exploration of their ethnic–racial identity (i.e., Asian American identity) and AARIIV. Consistent with ethnic–racial identity theories (Huguley et al., 2019; Umaña‐Taylor et al., 2014) and out‐of‐school activity frameworks (Eccles & Gootman, 2002; Kiyama et al., 2026; Williams & Deutsch, 2016), our findings underscore how organized activities can serve as key contexts for ethnic–racial socialization.

Ethnic–racial socialization occurred through both structured content and informal peer interactions. Some activities intentionally engaged students with Asian American histories, cultural practices, and community issues, such as celebrating cultural events and discussing Asian American experiences. Additionally, interacting with same‐ethnic–racial peers and having informal conversations fostered a sense of representation and belonging. Students shared cultural objects, such as heritage foods, and engaged in reflective conversations about their shared experiences. A recent systematic literature review on organized activities and ethnic–racial identity development (Kiyama et al., 2026) called for more research on how non‐community‐based activities and those not explicitly focused on race and ethnicity may shape adolescents' ethnic–racial identity development. Findings from this study address this concern demonstrating that, even when activities are not explicitly designed to promote ethnic–racial identity (i.e., Asian American identity) and exploration of racial identity ideological values (AARIIV), the social environment and peer dynamics within these activities can facilitate meaningful identity work, though it did not occur in all activities.

Another contribution of this study is the finding that students' understanding of AARIIV, including awareness of Asian American diversity and coalition‐building across ethnic–racial groups, emerged not only through structured programming but also through informal interactions with Asian American and non‐Asian American peers. Although prior research emphasizes the role of same‐ethnic–racial friendships in ethnic–racial identity development (e.g., Kao & Joyner, 2004; Kiang et al., 2010; Syed & Juan, 2012), this study shows that engagement with diverse peers and activity staff is also important for shaping ethnic–racial identity development, particularly AARIIV. Students articulated a growing awareness of shared struggles across racial and ethnic groups, reflecting commitments to coalition‐building and collective action. The current findings align with emerging research on the importance of cross‐group friendships for ethnic–racial identity development (Lorenzo et al., 2024) and underscore the value of organized activities as spaces for acknowledging diversity within one ethnic–racial group and fostering interracial solidarity across differing communities.

Notably, students' experiences were not always uniformly positive, and some of them highlighted the challenges that arose. For example, some students involved in activities with fewer Asian American peers and activity staff experienced microaggressions and discrimination, prompting critical reflection on their Asian American identity. This pattern suggests that simply being in diverse settings does not automatically lead to positive ethnic–racial identity development and that staff need to intentionally and actively foster these processes. Scholars have stressed the importance of being intentional in how activity staff approach diversity, fostering commonalities among adolescents while taking culturally responsive approaches that value diversity (Gutiérrez et al., 2017; Kiyama & Simpkins, 2026; Okamoto et al., 2012). When structured effectively, diverse activity settings may promote identity development by encouraging intercultural understanding (Kiyama & Simpkins, 2026; Lorenzo et al., 2024).

Overall, these findings highlight how organized activities provide ethnic–racial socialization not only through structured content but also through peer interactions and informal conversations. Exposure to diverse ethnic–racial peers, including both same‐ethnic and diverse Asian American peers as well as non‐Asian American peers, expands students' understanding of AARIIV and strengthens their commitment to solidarity across various ethnic–racial groups. As previous research emphasizes the positive impact of same‐ethnic–racial friendships on ethnic–racial identity development, this study underscores that interactions with peers of diverse ethnic–racial backgrounds are equally valuable for promoting collective consciousness and cross‐group understanding (Kiyama & Simpkins, 2026; Lorenzo et al., 2024; Okamoto et al., 2012).

#### Establishing a thriving activity environment

Our findings underscore that organized activities can better support ethnic–racial identity development when grounded in a positive activity environment. This pattern aligns with our prior work (Kiyama et al., 2026) emphasizing the broader, multidimensional nature of ethnic–racial identity as a meta‐construct. Extending this work, the present findings suggest that a thriving activity environment may also support the development of specific ideological values reflected in AARIIV. Students were more receptive to ethnic–racial socialization messages when they experienced warmth, support, and psychological safety within the activity setting (Kulish et al., 2019; Umaña‐Taylor & Hill, 2020). A thriving activity environment—characterized by respectful peer and staff relationships, inclusive practices, and emotional support—provided the foundation for students to engage in exploration of AARIIV.

Exploring AARIIV, particularly dimensions such as interracial solidarity and collective action, requires students to feel comfortable engaging in difficult conversations about race and inequality. This study supports the theoretical argument that, when students feel valued and respected, they are more likely to engage with complex and potentially challenging aspects of racial identity exploration (Umaña‐Taylor & Hill, 2020). For instance, students described feeling comfortable discussing issues of racial discrimination and coalition‐building with peers because the activity environment fostered openness and trust. The current findings also align with existing activity scholarship that emphasizes the importance of balancing ethnic–racial affirmation and cross‐group understanding (Simpkins et al., 2017; Okamoto et al., 2012). Activities that establish this balance not only promote positive racial identity development but also equip students with the skills and confidence to engage in broader social and political issues affecting their communities.

### Implications for practice and policy

This study offers important insights for organized activities seeking to support Asian American adolescents' positive development. Organized activities can serve as vital spaces for ethnic–racial identity exploration, especially in ways not typically available in formal school settings. Unlike classrooms, which are often constrained by standardized curricula and institutional structures, activities offer greater flexibility for youth‐driven discussions, culturally responsive programming, and relationship building that supports identity exploration (Halpern, 2002; McGovern et al., 2020).

As our findings presented, structured programming may provide meaningful opportunities for adolescents to engage with and explore their Asian American identity and AARIIV. For example, community‐based programs supporting Asian American adolescents have incorporated activities such as youth‐led explorations of local Asian American community histories, discussions of racial stereotypes such as the model minority myth, and cultural practices such as heritage language use, traditional arts, or storytelling about immigrant family experiences (Kiyama et al., 2026; Kiyama & Simpkins, 2026). Programming that offers ethnic–racial socialization allows students to connect their everyday experiences with broader historical and sociopolitical contexts related to Asian American communities.

Alongside structured programming, informal peer interactions represent another important avenue through which adolescents explore their ethnic–racial identities. As shown in our findings, identity‐related conversations can occur organically during informal moments with peers. In the present study, for example, students described bonding with peers over shared interests such as food and music, which they described leading to conversations about family traditions and cultural experiences. Practitioners may be able to support these informal interactions by intentionally designing activity environments that encourage peer engagement. Practitioners can structure break times to allow adolescents to socialize freely, arrange shared seating areas that promote conversation, or provide common spaces where adolescents can gather between activities. Such seemingly small design choices can create opportunities for adolescents to exchange everyday cultural experiences and learn from one another.

For adolescents to meaningfully engage with these socialization processes, organized activities should create a thriving activity environment that responds to adolescents' developmental needs and culture while affirming their sense of belonging through inclusive and respectful structures and practices (Simpkins et al., 2017; Ettekal et al., 2015). Prior research highlights several activity features that support a thriving activity environment, including providing physical and psychological safety, supportive relationships between staff and adolescents, clear yet flexible program structures, and positive social norms that encourage inclusivity and respect for all participating adolescents (see Kiyama et al., 2026 for further examples). Activities can further strengthen these environments by incorporating adolescent voices into program design. Although many students in this study reported that their activities supported their exploration of Asian American identity and AARIIV, others expressed mixed views or offered suggestions for improvement. Adolescents bring unique perspectives and experiences, which shape how they interpret and respond to the ethnic–racial socialization embedded within the activity (Simpkins et al., 2017; Williams & Deutsch, 2016). Some community‐based programs serving Asian American youth have tried addressing this issue by establishing youth advisory boards or regularly collecting participant feedback to guide program development (Kiyama & Simpkins, 2026). Incorporating adolescent input allows activities to ensure that programming remains responsive to adolescents' needs and interests.

Equipping activity staff with clear guidance on cultural responsiveness and effective strategies to meet adolescents' needs is also crucial to sustaining these efforts (Gutiérrez et al., 2017; Simpkins et al., 2017). Even when activity staff recognize the importance of cultural responsiveness, many feel unprepared to address culture‐related incidents (e.g., discrimination) or facilitate discussions on systemic injustices relevant to adolescents' lived experiences (Gutiérrez et al., 2017; Skelley et al., 2022). Staff may encounter situations in which adolescents share experiences of racial discrimination at school or express uncertainty about navigating cultural expectations between family and peers (Kiyama et al., 2026). Professional development opportunities that include case‐based discussions, role‐playing responses to microaggressions, and guided reflection on cultural assumptions may help staff feel more confident facilitating these conversations and supporting Asian American adolescents' identity exploration.

Overall, these findings highlight the importance of balancing structured and informal socialization, creating inclusive and culturally responsive thriving activity environments, equipping activity staff to meet adolescents' needs to support their exploration of ethnic–racial identity and AARIIV. By thoughtfully designing organized activities around these principles, practitioners can create spaces that support Asian American adolescents' ethnic–racial identity development while also fostering belonging and broader interracial solidarity and collective action.

### Limitations and future directions

Although this study provides valuable insights into the role of organized activities in shaping Asian American college students' Asian American identity and AARIIV, several limitations should be noted. First, this study did not use experimental data to assess the directionality of the relations between organized activity participation, Asian American identity, and AARIIV. Given that ethnic–racial identity development becomes particularly salient in adolescence and into early adulthood, it is possible that Asian American college students in this study perceived stronger links between their activity experiences and their ethnic–racial identity including AARIIV due to their current developmental stage (Azmitia et al., 2008; Umaña‐Taylor et al., 2014; Yoo et al., 2021). Collecting data measuring adolescents' level of their ethnic–racial identity and AARIIV pre‐ and post‐activity participation would enable a longitudinal examination of the association between activity participation and ethnic–racial identity development.

Second, Asian American college students reflected retrospectively on their high school activity experiences, which may introduce recall bias. Retrospective narratives do not represent objective recordings of past events but rather individuals' present‐day interpretations of those experiences (McAdams, 2001). At the same time, recalling experiences during early adulthood may allow individuals to connect past experiences to their current beliefs, values, and identities through greater thematic coherence (Habermas & Bluck, 2000; McAdams, 2001). In this way, these accounts provide insight into how individuals make meaning of their experiences as developmental processes are shaped in part by how contexts are perceived and interpreted (Eccles & Wigfield, 2020). Additionally, prior work suggests that youth may benefit from learning AARIIV through programs and interventions (Yoo et al., 2021). Gaining insight from college students' reflections can help clarify how these values are understood and experienced, which may inform the design of developmentally appropriate approaches for adolescents. Given the complexity of reflecting on race, ethnicity, identity, and ideological values rooted in sociopolitical awareness, being an emerging adult may facilitate more elaborated accounts of these processes. Simultaneously, understanding how these processes unfold during adolescence remains important. Thus, while retrospective accounts provide insight into how Asian American college students interpret the role of organized activities in their identity development, future research following adolescents in real time could complement these findings by capturing identity processes as they unfold.

Third, the study sample primarily reflected the experiences of U.S.‐born Asian American college students who grew up in Southern California, offering a focused lens on ethnic–racial identity development within a particular sociocultural context. Rather than emphasizing generalizability, these findings provide insight into how identity processes unfold in settings with a large and historically established Asian American population, where adolescents may have increased access to identity‐relevant resources, such as same‐ethnic–racial peers, culturally affirming programs and institutional support, which may facilitate greater opportunities for ethnic–racial exploration and affirmation (Chan, 2017; Umaña‐Taylor & Shin, 2007; Wong, 2023). Southern California represents a unique context shaped by a long history of Asian American immigration, community formation, and activism, as well as place‐specific racial dynamics that influence how racial meanings are constructed and negotiated (Cheng, 2013; Syed et al., 2018). At the same time, this work highlights the importance of extending research to more fully capture the diversity of Asian American adolescents' experiences across contexts and social positions. Future research would benefit from examining how these processes unfold in settings where Asian American adolescents are a numerical minority, where identity development may be shaped by increased visibility, marginalization, or intergroup comparison processes (Sellers et al., 1998; Tajfel & Turner, 1986). Additionally, extending this work to more fully capture the experiences of first‐generation Asian American adolescents who immigrated during childhood or adolescence would further enrich understanding how migration history and generation status shape ethnic–racial identity development (Feliciano & Rumbaut, 2019; Umaña‐Taylor et al., 2014). Furthermore, future studies that include more gender‐diverse samples could illuminate how gendered and racialized experiences interest to shape identity development, particularly given the emerging evidence that Asian American women may experience racialized and gendered socialization and discrimination in distinct ways (Ahn et al., 2022; Mukkamala & Suyemoto, 2018).

Finally, the study focused primarily on student experiences within organized activities, but it did not examine the broader contextual factors that may shape these experiences. Family socialization practices, school climate, and neighborhood characteristics likely interact with activity experiences to influence identity development (Umaña‐Taylor et al., 2014). Additionally, the current findings highlight processes with organized activities aligning with the study goals, but it is important to acknowledge that activities are only one context within the broader ecology of racial socialization. Racial socialization experiences occur beyond the settings of organized activities (e.g., on their way to school, daily interactions with peers outside of activities). Future research could complement the current findings using a broader ecological approach to explore how these intersecting layers of influence—such as organized activities, families, peer networks, and school policies—shape racial identity development.

## CONCLUSION

Findings from the present study underscore the potential importance of organized after‐school activities in fostering ethnic–racial identity development among Asian American college students, particularly in their exploration of AARIIV. The structured programming and informal peer interactions in activities create meaningful spaces for identity exploration. A thriving activity environment built on respect and inclusivity encourages students to engage with complex topics like racial discrimination and interracial solidarity. The current findings not only contribute to the theoretical understanding of ethnic–racial socialization but also provide practical insights for organized after‐school activities of all types to support and affirm Asian American college students' ethnic–racial identity.

## AUTHOR CONTRIBUTIONS

**Fuko Kiyama:** Conceptualization; investigation; funding acquisition; writing – original draft; visualization; methodology; validation; data curation; formal analysis; project administration; writing – review and editing; software; resources. **Sandra D. Simpkins:** Writing – review and editing; supervision; resources.

## FUNDING INFORMATION

This review was supported by the University of California, Irvine, Graduate Dean's Dissertation Fellowship and Deborah Lowe Vandell Research Award awarded to the first author.

## CONFLICT OF INTEREST STATEMENT

The authors declared no potential conflicts of interest with respect to the research, authorship, or publication of this article.

## ETHICS STATEMENT

This study was determined to qualify for Exempt Self‐Determination under HRP Policy 12 at the University of California, Irvine. The determination has been registered with the Human Research Protections (HRP), and UCI IRB review was not required.

## CONSENT TO PARTICIPATE

In line with ethical research standards, informed verbal consent was obtained from all participants, participation was voluntary, and data were collected and reported in a manner that ensured confidentiality and minimized potential risk.

## Supporting information


Figure S1.

## Data Availability

Data are available from the corresponding author upon reasonable request.

## APPENDIX A

### A.1 Semistructured interview transcript

**Table:**

**Participant ID:**	**Interviewer:**
**Pseudonym:**	**Date:**

### A.2 INTERVIEWER INSTRUCTION

1. Before starting the interview, ask the participant to pick a pseudonym or a nickname that you would call them during the interview so that their identity remains private. If the participant struggles, recommend a best friend's name or perhaps a favorite TV or movie character
2. Ensure the participant feels comfortable and let them know that you will be audio recording the interview
3. Note the following color codes: **BLACK**—INTERVIEW QUESTIONS, ***BLUE*** —INTERVIEWER STATEMENTS, **RED**—INTERVIEW INSTRUCTIONS

### A.3 INTRODUCTION

Thank you for taking some time to talk to me today. During this interview, I will be asking several questions about your experiences in high school, including your thoughts on being Asian American. We will also be talking about the role that organized after‐school activities might have had in developing or learning more about being Asian American in the United States. Everything you say during the interview will be kept confidential and we will not report to anyone what you individually say. Your participation in this interview is voluntary. You don't have to answer any question that makes you uncomfortable and you can stop this interview at any time if you decide you no longer want to participate. Do you have any questions before we start with the interview?

Start the recorder and say the following before starting the interview.

My name is [name of interviewer] and I am here with [participant's pseudonym]. Her/his/their ID number is [#] and it is [date].

### A.4 WARM UP

I wanted to ask a few questions first to get to know you more as a student when you were in high school.

1. Can you describe yourself as a student in high school?
2. What were your academic interests in high school?
3. What were your extracurricular interests in high school?

### A.5 SECTION 1: GENERAL QUESTIONS ABOUT BEING ASIAN AMERICAN

I next want to learn a little bit more about your racial and ethnic identity. These next questions will ask about your thoughts on the term “Asian American” and your experiences as an Asian American high school student.

1. How do you identify yourself racially and ethnically?
2. What do you consider the most important part of your racial and ethnic identity?
3. [If they have not mentioned identifying as Asian American] Do you identify as Asian American?
4. How did you feel about the term “Asian American” in high school?
5. Who did you consider as Asian American in high school?
  - [If they do not mention multi‐ethnic Asian Americans] How about Asian Americans who share multiple memberships in different Asian ethnic groups?
6. Did your perception of the term Asian American change during high school?
  - [If yes] How did it change?

### A.6 SECTION 2: EXPERIENCE IN ORGANIZED AFTER‐SCHOOL ACTIVITIES

I would like to learn more about your experience participating in organized afterschool activities in high school. To be on the same page, I wanted to provide a definition of what I consider as organized activities. Organized after‐school activities are defined as activities that have at least one adult leader and meet at regularly scheduled times. These include being involved in sports teams, arts programs (e.g., dance, music), school clubs, after‐school programs, and community center programs (e.g., Boys & Girls Club). They can be located at schools or in the community (e.g., community center, church).

Once the participant has the same understanding of what organized afterschool activities mean, start asking the questions below.

#### A.6.1. General experiences

1. What are the organized activities you participated in high school?
2. Can you share your overall experience participating in organized after‐school activities in high school?
  - [If they do not mention much] Can you share any positive experiences you had participating in organized after‐school activities in high school?
  - [If they do not mention much] Can you share any negative experiences you had participating in organized after‐school activities in high school?
3. What motivated you to participate in those activities in the first place?
4. Could you describe the organized activities that made an impact on your understanding of being Asian American or [insert label they use to identify themselves]?
  - Could you describe the race/ethnicity of activity staff and the students in [this activity/these activities]?
  - What was your relationship like with activity staff in those activities?
5. Were there other things outside of school, besides the organized activities, that made an impact on understanding what it means to be Asian American or [insert label they use to identify themselves]?
  - Could you further describe what those things were and why they made an impact?
  - [If the impact was positive] Why did those things make a positive impact?
  - [If the impact was negative] Why did those things make a negative impact?
6. In general, what are the types of spaces that have made you feel positive about your racial/ethnic identity?
  - Could you further describe why those spaces made you feel positive about your identity?
7. What are the types of spaces that have made you feel less positive about your racial/ethnic identity?
  - Could you further describe why those spaces made you feel less positive about your identity?

#### A.6.2. Culturally responsive program features

Culturally responsive practices refer to approaches, strategies, or actions that take into account the cultural backgrounds, identities, and experiences of individuals or communities. It means showing respect for the unique backgrounds and identities of people from different cultures. Examples of being culturally responsive are incorporating different cultural traditions, connecting community and family knowledge, and being mindful of the communication styles and preferences of participants from different cultural backgrounds.

For the next set of questions, I will ask about the culturally responsive program features that you experienced in your organized afterschool activities. Based on the previous questions that you answered regarding your participation in organized afterschool activities, please choose one activity that positively impacted your understanding of being Asian American to answer the next set of questions.

Give some time for the participant to choose the organized after‐school activity that they will talk about in the next set of questions. Once they are ready, ask the questions below.

1. Could you describe the organized after‐school activity that you will be talking about in this set of questions?
  - Why did you join [name the organized after‐school activity]?
  - How long did you participate in [name the organized after‐school activity]?
  - What were the demographics (e.g., race/ethnicity) of participants at [name the organized after‐school activity]?
  - What were the types of things you did at [name the organized after‐school activity]?
2. Could you provide examples of what the staff or adults in your activity did that you consider responsive to your needs?
3. Could you provide examples of what the staff or adults in your activity did that you consider responsive to your ethnic and racial background?
  - How have these actions by the activity staff impacted you?
4. Were there other things besides what the activity staff said or did in this activity that have been particularly responsive to your needs and experiences?
  - [If there is an answer] How have these positively impacted your experience as a participant?
5. Have you encountered any challenges or areas where the organized afterschool activity could have been **more** culturally responsive to the participating students?
  - [If yes] What improvements or changes would you suggest to enhance the cultural responsiveness of this activity?
6. Did your organized afterschool activity provide opportunities to talk about your race and other racial groups in the United States?
  - [If yes] Could you further describe what the activity staff said or did to create that space and how often these opportunities occurred?
  - How did you and other participants feel when these conversations occurred?
  - Do you agree with how they provided the opportunity to talk about these topics? [If not], could you explain why and what you would have wanted instead?
7. From your perspective, did your organized afterschool activity have a social justice‐oriented focus [if the participant has a hard time understanding social justice‐oriented examples, mentioned discussions about systemic racism, activism‐related activities]?
  - [If yes] Could you further describe what the activity staff said or did that made you aware that they had a social‐justice‐oriented focus?

### A.7 SECTION 3: SUPPORT IN EXPLORING ASIAN AMERICAN RACIAL IDENTITY

This is the last section of the interview. The next set of questions will ask about your experience in organized afterschool activities during high school and how they may have helped you explore your Asian American racial identity. I want you to reflect back on the organized activity that you described in the previous section when you answer the next set of questions.

1. How, if at all, did participating in [name of organized activity] support your understanding of what it means to be Asian American?
  - Can you share specific examples or aspects of the activity that was particularly impactful?
2. Did [name of organized activity] provide opportunities for interracial solidarity and learning about the struggles of other communities of color (e.g., non‐White communities such as African, African American, Asian, Latino, Middle Eastern and North African, Native American, Pacific Islander, and Slavic)?
  - [If yes] How has this opportunity impacted your understanding of issues that surround communities that are not Asian American?
3. Did [name of organized activity] provide opportunities to learn about Asians overseas?
  - [If yes] How has this opportunity impacted your understanding of issues involving Asians overseas?
4. Looking back, is there anything you wished [name of organized activity] could have done to better support your exploration of Asian American racial identity?

That would be all the questions I prepared for this interview. Is there anything else I should have asked you, or is there anything else you would like to share about this topic?

[If the participant has no other comments to add]. Great, I will now turn off the recording.

Turn off the recorder and say the following to wrap up the interview.

Thank you again for taking the time to talk with me today. I sincerely appreciate your participation in this study. We will now have you complete the post‐interview survey.

[Insert the post‐interview survey link into the chat box].

*Please let me know when you have completed the survey*.

When the participant indicates that they completed the survey, check the response Excel sheet and make sure that it was completed.

Thank you for completing the post‐interview survey. We have now completed your participation in this study. While your interview is now complete, please feel free to reach out to me should you have any questions or concerns. [The first author], our lead researcher will be following up with you within the next week to send you the $10 gift card. She will be sending the gift card using the email we have been using to communicate with you.

End of interview + post‐interview survey.

## APPENDIX B

### B.1 Post‐Interview survey

Thank you very much for participating in the interview! Before we conclude your participation in the study, please fill out the following questions that will provide further context for our study.

* Indicates required question

1. Demographic Information
  - What is your gender? * Mark only one.
  - Female
  - Male
  - Non‐binary
  - Other
  - Prefer not to answer
2. What year are you in college? * Mark only one.
  - First year·
  - Second year
  - Third year
  - Fourth year
  - Other

2a. If you answered “other” in question 3, please indicate your year

3 What is your parents' highest level of education? *
4 Were you born in the United States? *Mark only one.
  - Yes
  - No
  - Prefer not to answer

4a. If you answered, “No,” for question 3, at what age did you arrive in the United States?

### B.2 PARTICIPATION IN ORGANIZED AFTER‐SCHOOL ACTIVITIES

Organized after‐school activities are defined as activities that have an adult leader and meet at regularly scheduled times. These include being involved in sports teams, arts programs (e.g., dance, music), school clubs, after‐school programs, and community center programs (e.g., boys & girls club). They can be located at schools or in the community (e.g., community center, church).

1. Please indicate the type of organized after‐school activities you participated in during high school.* Check all that apply.

- Sports (e.g., team sports, individual sports)
- Arts (e.g., theater, dance, music)
- Academic (e.g., year‐book, debate, chess)
- Religious (e.g., faith‐based youth groups)
- Cultural (e.g., clubs focused on learning about a specific culture)
- Other

1a. If you answered, “Other,” in question 2, please indicate the type of organized after‐school activity.

Survey was conducted via Google Forms. Students were required to answer the questions with an asterisk.
