# Supplementary material for: Organized after‐school activities and Asian American high school students' Asian American identity and Asian American racial identity ideological values
Source: J Res Adolesc. 2026 Aug 21;36(3):e70249. doi: 10.1111/jora.70249 (PMC13494687; doi:10.1111/jora.70249)
Supplement: Supplementary file 1 [file JORA-36-0-s001.docx]

**Supplementary Material**

**Figure S.1**

*Proposed Framework by Which Organized After-School Activities Can Promote Ethnic-Racial*

*Identity Development*

*Note.* ^1^Umaña-Taylor et al. (2014), ^2^Eccles and Gootman (2002); Kiyama et al., 2026, ^3^Williams and Deutsch (2016).

**Figure S.2**

*Color* *Chart Visualizing Students’ Activities and Association with Asian American identity and Asian American Racial Identity Ideological Values*

*Note.* Participants’ names are pseudonyms. Under gender, F indicates female, and M indicates male. The numbers in each cell represent the total number of activities within each activity type that was either (1) associated with Asian American identity (indicated in the color orange), (2) associated with Asian American racial identity ideological values (indicated in the color blue), or (3) associated with both Asian American identity and Asian American racial identity ideological values (indicated in the color purple). For example, Emily has a 1 under both sports and cultural, indicating she participated in one sport activity that was associated with her Asian American racial identity ideological values and one cultural activity that was associated with her Asian American identity. The names of the activities are listed on the right side of the color chart. The letter in parentheses following each activity name denotes the activity type: S = sports, A = arts, R = religious, C = cultural, V = volunteer and service, L = leadership.
